# Cells Special Issue: “The Molecular and Cellular Basis of Retinal Diseases”

**DOI:** 10.3390/cells12151933

**Published:** 2023-07-26

**Authors:** Steven J. Pittler, Steven J. Fliesler

**Affiliations:** 1 Department of Optometry and Vision Science, Vision Science Research Center, School of Optometry, University of Alabama at Birmingham, Birmingham, AL 35294-0019, USA; 2 Departments of Ophthalmology and Biochemistry and Neuroscience Graduate Program, Jacobs School of Medicine and Biomedical Sciences, The State University of New York-University at Buffalo, Buffalo, NY 14203-1121, USA; fliesler@buffalo.edu; 3 VA Western NY Healthcare System, Buffalo, NY 14215-1129, USA

The recent success in the treatment of hereditary retinal disease caused by defects in the RPE65 gene and the FDA approval of this treatment has established the importance of the study of animal models and the translational impact of these research findings. This success has sparked an intense interest in the development of animal models for other hereditary eye diseases and has led to a dramatic increase in clinical trials in this area. This Special Issue focuses on studies involving new animal models of human hereditary retinal degenerations and highlights the current knowledge in the field regarding the basic metabolic processes and biochemical pathways that offer promising new therapeutic targets for successful intervention in such retinal diseases. Here, we provide a brief summary of each of the 15 articles included in this Special Issue, the first 7 of which are original research articles, and the following 8 are review articles.

First, Qi and coworkers [1] present their findings that demonstrate impaired diurnal rhythmicity of autophagy in mouse and rat models of diabetic retinopathy (DR), compared with normal controls. Using immunohistochemistry, they monitored the expression and spatial distribution of autophagy marker proteins (“Atgs”: Atg7, Atg9, LC3, and Beclin1) as a function of the light–dark cycle. The Atgs were expressed in both neuronal as well as endothelial cells in the retina and exhibited distinct patterns of expression, which were subject to diurnal variability. The expression patterns were phase-shifted, and their diurnal rhythmicity was disrupted in diabetic animals, compared with normal controls. The authors suggest that the restoration of the diurnal rhythmicity of expression of Atgs may provide a new avenue for therapeutic intervention in DR.

Ramachandra Rao et al. [2] describe the initial characterization of a newly developed mouse model of retinitis pigmentosa-59 (RP59; OMIM #613861), an autosomal recessive form of RP that involves progressive, irreversible dysfunction, and degeneration of retinal rod and eventually cone photoreceptors. The most prevalent form of this disease involves a K42E point mutation in the enzyme dehydrodolichyl diphosphate synthase (DHDDS), which, along with its binding partner Nogo-B receptor (NgBR), comprises the *cis*-prenyltransferase (CPT) enzyme complex. CPT is required for the synthesis of polyprenols and dolichols; in turn, the phosphorylated derivatives of dolichol are required for protein N-glycosylation and other forms of glycosylation in the cell. Using CRISPR/Cas9 technology, a homozygous K42E DHDDS knock-in mouse model was generated (*Dhdds^K42E/K42E^*) on a C57Bl/6J background. Surprisingly, the phenotype was very mild (unlike the human disease): There was no overt retinal degeneration, and there was no evidence of compromised protein N-glycosylation, even up to one year of age. Nevertheless, the expression of glial fibrillary acidic protein (GFAP) was markedly upregulated in a pattern indicative of gliotic reactivity (involving astrocytes and Müller glia). Such a glial response typically is associated with disturbances in the neuronal environment and frank neuronal degeneration. While further characterization of this novel RP59 mouse model is warranted, these initial findings cast doubt on the current classification of RP59 as a “congenital disorder of glycosylation” (CDG) and suggest that the underlying disease mechanism is more complex than simple loss-of-function of DHDDS activity.

A follow-up, related report [3] involves the generation and initial characterization of another mouse model of RP59, where DHDDS was selectively ablated in retinal pigment epithelial (RPE) cells, using Cre-lox technology. A global floxed *Dhdds* mouse line (*Dhdds-^flx/flx^*) first was generated, and those mice were mated with mice expressing Cre recombinase under the control of the VMD2 (vitelliform macular degeneration 2) promoter to affect the RPE-targeted deletion of *Dhdds*. Both lines were generated on a C57Bl/6J background. RPE-specific Cre expression was confirmed by crossing VMD2-Cre line mice against a ZsGreen reporter mouse line. Progressive, but “patchy”, RPE and neural retina degeneration was observed over the course of a three-month postnatal observation, starting by about the first postnatal month, including a geographic atrophy-like phenotype, with correlative electrophysiological deficits impacting rod- and cone-driven photoresponses. It was concluded that the dysfunction and degeneration caused by the loss of DHDDS expression in the RPE provoked the underlying photoreceptor cells to degenerate in response over a few months. Unfortunately, neither the status of protein glycosylation nor the quantification of dolichols in the RPE or neural retina of this mouse line was assessed. However, presumably, the ablation of the *Dhdds* gene in the RPE would have prevented the synthesis of new dolichol molecules, starting around the onset of Cre recombinase activity, which in turn would be expected to significantly reduce or prevent protein N-glycosylation. Further characterization of this RP59 mouse model is clearly warranted.

Hollingsworth and Gross [4] studied heterozygous and homozygous Ter349Glu rhodopsin mutant mice (an RP model) in comparison to age-matched wildtype (WT) control mice, using immunohistochemistry, electroretinography (ERG), optical coherence tomography (OCT), and fluorescein angiography (FA). Along with the expected progressive retinal degeneration, including dysfunction, degeneration, and loss of photoreceptors, they found evidence for the involvement of both the innate and the autoimmune systems in the underlying mechanism of the mutation-induced retinal degeneration phenotype. Among the several observations, they found an increase in citrullination in mutant retinas, compared with WT controls, which could contribute to an autoimmune response in the retina. While not discussed, this also may be indicative of increased oxidative stress (which has been implicated in a variety of ophthalmic diseases, especially hereditary retinal degenerations). Overall, these findings suggest that therapeutic interventions for hereditary retinal degenerations, particularly the various forms of RP, should take into consideration the modulation of both innate and autoimmune mechanisms.

Wolk et al. [5] provide new experimental data, derived from both human specimens, cultured ARPE-19 cells, and mouse studies, regarding the mechanism underlying Sorsby’s fundus dystrophy (SFD), which is a rare macular dystrophy involving chronic choroidal neovascularization (CNV) that leads to progressive vision loss. SFD is caused by mutations in the tissue inhibitor of the metalloproteinase (MMP)-3 (*TIMP3*) gene and has some phenotypic features similar to those of age-related macular degeneration (AMD). The authors report that levels of glycosaminoglycan hyaluronan (HA) were elevated in plasma and RPE/choroid specimens from AMD patients. This finding was also observed in the case of mice harboring the S179C-TIMP3 mutation. The human-derived ARPE-19 cell line expressing the S179C-TIMP3 mutation also exhibited HA accumulation, compared with ARPE-19 cells expressing wildtype TIMP3. They also report that FGF-2 (fibroblast growth factor) induced the accumulation of HA in RPE cells. Taken together, these findings tend to implicate the involvement of the TIMP3-FGF-HA axis in the pathogenesis of CNV in SFD, as well as possibly in AMD.

Asare-Bediako et al. [6] characterized the retinal phenotype associated with the high-fat (60% fat; HFD) mouse model of obesity, in comparison to mice raised on a low-fat (10% fat; LFD) diet for one year. Both models were generated on a C57Bl/6J background. The HFD diet provoked hypercholesterolemia, reduced insulin sensitivity index, and increased body mass, compared with the LFD cohort, but not hyperglycemia (unlike type 2 diabetes models). Scotopic (but not photopic) ERG a- and b-wave amplitudes were reduced within 6 months in the HFD cohort, compared with the LFD cohort, and by 12 months, mice on the HFD diet exhibited increased retinal nerve infarcts and vascular leakage, as well as reduced vascular density, but without an increase in acellular capillaries compared with mice on the LFD regimen. Curiously, by 12 months, there were no differences in ERG responses comparing the two dietary groups; nevertheless, in the LFD cohort, when comparing the data obtained at 6 months vs. 12 months of the study period, both scotopic and photopic ERG amplitudes were reduced at 12 months. The authors concluded that the HFD mouse model has value for understanding the effects of prediabetes and hypercholesterolemia on the retina. The HFD-induced changes manifested more slowly than those observed in type 2 diabetes models (e.g., the *db/db* mouse), and neuronal damage (as evidenced by ERG deficits) was observed prior to vascular changes, consistent with what was observed in human diabetic patients. They also assessed various biomarkers and retinal function in mice raised on the so-called “Western diet” (WD), which has lower fat content than the HFD regimen and does not induce hyperglycemia. They found that the WD induced elevated GFAP expression in subpopulations of Müller glia (vimentin-positive cells) but not in the retinas of LFD mice. They also observed an increased expression of HIF-1α in some (but not uniformly in) vascular endothelial cells in the retinas of mice in the WD cohort but not in those of mice in the LFD cohort, suggesting some degree of perhaps regional hypoxia induced by the WD regimen. Liver X receptor *beta* (LXRβ) levels, which tend to correlate with lipid deposition, were also reduced in the retinas of mice in the WD cohort, compared with the LFD cohort. Initially (by 3 months of WD treatment), this reduction was only evident in the ganglion cell layer; however, by 6 months, the reduction was also apparent in the inner and outer nuclear layers (INL and ONL), compared with mice in the LFD cohort.

Voigt et al. [7] present a case report of a 70-year-old patient with retinal degeneration attributed to autoimmune retinopathy (AIR) in comparison with specimens from four unaffected human eye donors. They employed single-cell RNA sequencing on foveal and peripheral retina samples to evaluate cell-specific gene expression differences between normal vs. degenerating retinas to evaluate how different populations of retinal cells respond to photoreceptor degeneration. Genes were assigned clusters, and cell types were determined based on the expression of unique cell-type specific markers (e.g., the cluster exhibiting high expression of the Müller glia markers RLBP-1 (retinaldehyde-binding protein 1) and CRALBP1 (cellular retinaldehyde-binding protein 1)). Distinct populations of glial cells with an expression profile consistent with reactive gliosis (e.g., GFAP and the annexins ANXA1 and ANXA2) were identified in the sample from the retinal degeneration patient. Notably, the expression patterns of other inner retina cell types were remarkably similar when comparing normal vs. degenerated retina samples. The authors conclude that this provides evidence that glial cells have a distinct transcriptome in the biological context of retinal degeneration, as compared to a normal healthy retina, and that this provides insight into the responses of the retina to a blinding inflammatory condition (in this case AIR) at the cellular and transcriptional levels.

Nashine and Kenney [8] review the role of mitochondrial dysfunction in the etiology of age-related macular degeneration, focusing on mitochondrial-derived peptides (MDPs) produced from the 12S and 16S mitochondrial rRNAs. These peptides regulate cell survival and growth or are critical for the regulation of muscle and fat metabolism and the prevention of hepatic steatosis. In AMD, mitochondrial damage in the retinal pigment epithelium (RPE, a single layer of cells posterior to the retina) leads to RPE dysfunction, which in turn, leads to retinal dysfunction due to the many functions the RPE performs that supports and protects the retina. Because of the protective functions of MDPs, they show promise for the treatment of AMD and other disorders due to mitochondrial dysfunction. The role of specific MDPs such as humanin, and small human-like peptides (SHLPs) is discussed, as well as the potential to use these peptides for the treatment of AMD. Additionally, an excellent review of mitochondrial function is provided.

Collin et al. [9] present a comprehensive list and review of mouse models of retinal degeneration based on entries in the PubMed and Mouse Genome Informatics databases. The review is focused on monogenic disorders that exhibit photoreceptor cell loss, revealing a wide range of onset of degeneration and the rate of cell loss. The associations between functional changes observed in ciliary function, DNA repair, and cellular chloride homeostasis with retinal degeneration genes are reported. Surprisingly, the reviewed mouse models represent only 40% of the currently known spectrum of Inherited Retinal Degeneration based on the RetNet compendium of human IRD. This work represents a major undertaking to provide a comprehensive resource of monogenic mouse models of IRD that will be useful to help guide translational studies and support the development and characterization of the remaining 60% of IRD that remains to be modeled.

Winkler et al. [10] review advances in studies of large animal models of inherited retinal degeneration (IRD). While the main focus is on dog models, disease modeled in pigs, cats, and horse is also reviewed for some diseases. The reviewed animal models represent defects in almost 30 genes that affect phototransduction, photoreceptor development, the visual cycle, signal transmission, channelopathies, structural integrity, and other aspects of retinal homeostasis. As these large animals have retinas closer in size to the human eye and very similar physiology, they provide superior models to study the disease they are modeling and to test possible treatments.

Wensel [11] reviews the role of phosphoinositides (PIs) in the retina and retinal pigment epithelium (RPE) and their role in retinal disease. This review covers chemical structures and membrane content and dynamics, and the importance of the membrane for trafficking and other functions. PIs play an important role in ciliary function for membrane trafficking and movement. The regulation of PI metabolism by light in the retina is discussed, and although light clearly affects this process, a role for PIs in the regulation of phototransduction, akin to what is observed in *Drosophila*, is not supported by current studies and the kinetics of the process, which can be explained without a need for PI metabolism. Disease states associated with seven different genes encoding the proteins involved in PI metabolism are reviewed. The gene products function in membrane trafficking and sorting, (PI metabolism (phosphatases) and PI transfer proteins), and other proteins with PI-binding domains. While knowledge in the field has progressed significantly, there is still much to learn about the role of PIs in the retina and RPE.

Tebbe et al. [12] describe the progress in the study of peripherin 2 (Prph2), a structural protein required for the formation of rod photoreceptor outer segment disks, thus far. This review focuses on the knowledge gained from studies of mouse models and related patient studies and provides a brief summary of gene therapy applied to some of the mouse mutants that have been created. In total, studies on 14 Prph2 mouse strains and 9 patient mutations are reviewed and compared, providing insight into the role of the protein in outer segment morphogenesis and pathophysiology. These studies show that Prph2 complexes with another protein ROM1 are critical for proper disc formation. Other important findings that are reviewed include the importance of Prph2 tetramers, higher-order complexes versus the role of Prph2/Rom1 complexes, and the differences in the role of Prph2 in the rods and cones. Studies using established adeno-associated viruses or nanoparticles for gene delivery to mouse retina both resulted in a significant degree of structural and functional recovery but not back to WT levels. A better understanding of second-level complexities of protein oligomerization and its role in rod and cone function is needed to better direct effective gene therapies.

Picard et al. [13] provide a comprehensive review of the importance of iron (Fe) and iron-associated proteins in the retina in health and disease. Oxidative state changes from Fe^2+^ to Fe^3+^ can be the difference between normal and disease states. The authors review 27 iron-metabolism-related proteins and 14 knockout rodent models. After a brief, but very well-developed review of retina structure and oxygen distribution, general and retina iron homeostasis in all major retinal cell types is discussed. A key point of the review is that many of the main proteins involved in iron homeostasis are locally synthesized in the retina, indicating a separate system of iron control distinct from systemic. Next, the distribution and roles of iron homeostasis in different retinal cell types and layers are elucidated. Iron’s potential role as a biological sensor for molecular oxygen and nitric oxide, as well as its role in DNA synthesis and repair, cell proliferation, oxygen transport and regulation, and visual function, is discussed. In visual function, RPE65, which is essential for the regeneration of the chromophore 11-cis retinal in rods, is an iron-dependent enzyme. Iron, like vitamin-A and its derivatives, must always be chelated to some protein, usually ferritin. Free iron is a known or suspected cause of many disorders, such as oxidative stress, inflammation, angiogenesis, siderosis and retinal hemorrhaging, iron accumulation disorders, age-related macular degeneration, diabetic retinopathy, glaucoma-mediated neuropathy, and inherited retinal disease, and the mechanisms of each of these disorders are considered. Lastly, therapeutic intervention through the use of iron chelation is shown in different models of the perturbation of iron metabolism.

Matteis and Rizzello [14] review nanoparticle-mediated delivery of biomolecules to the retina with a focus on metal and polymeric lipid-based technologies. After a brief review of ocular anatomy and common ocular disorders, the authors focus on the use of metals (gold or silver) that have been used by many different groups trying to develop safe and efficient delivery systems to the retina. Many possible routes of administration are considered, along with the potential complications for each route. Over 40 published works using silver or gold nanoparticles (NPs) for delivery are reviewed in terms of efficiency of delivery, adverse effects, and outcome evaluation. After highlighting the distinction between inorganic, non-degradable, bio-inspired nanoparticles and degradable lipid-based or chitosan/alginate-based NPs, the authors provide a review of over 50 reports concerning the use of these delivery vehicles. Both types of NPs have been successful in gaining access to many parts of the eye and show varying degrees of efficacy in treating a wide range of disorders. Exciting new technology based on active and self-propelling NPs that can move through the tissue to target locations is compelling due to the low toxicity that was observed and the ability to control the movement well beyond the range of simple diffusion. The authors conclude with the promise and potential of nanotechnology and the realization that the field is still in its infancy and will need much more development to be realized as the go-to tool for the treatment of many ocular disorders.

Sinha et al. [15] remind us of the importance of understanding basic metabolism and the interaction among various pathways operating in the body. This review focuses on the importance of serine and its many metabolites that are critical for diverse functions in all cells, including the retina. However, this review extends well beyond the basic metabolic needs of all cells, homing in on key actions of serine that are critical for the neural retina and RPE. In addition to being a fundamental building block of protein, serine is required as a precursor to other amino acids (cysteine, glycine, and methionine) and sphingolipids. Tissues that have requirements for serine beyond what can be provided through the blood have their own synthesis machinery to produce serine, including retina tissue and RPE. Not only is the protein enantiomeric form of serine (L-serine) needed, but so is D-serine, which supports synaptic function. Decreased serine levels are implicated in the etiology of macular telangiectasia type 2, diabetic retinopathy, and inherited retinal degeneration. Serine has a role in the epigenetic regulation of gene expression due to its role in the synthesis of the universal methyl donor, S-adenosyl methionine, which is also required for protein isoprenylation. Serine can act as an antioxidant and inflammation mediator due to its role in the production of glutathione. It also has a role in the regulation of phagocytosis of photoreceptor disks through the action of phosphatidylserine. The authors conclude with the significant potential for disease intervention using serine-based small molecules, providing an example of the use of D-serine in the treatment of diabetic retinopathy.
